# Incidence and predictors of tuberculosis among adult people living with human immunodeficiency virus at the University of Gondar Referral Hospital, Northwest Ethiopia

**DOI:** 10.1186/1471-2334-13-292

**Published:** 2013-06-28

**Authors:** Kefyalew Addis Alene, Ansha Nega, Belaynew Wasie Taye

**Affiliations:** 1 Department of Health Officer, Institute of Public Health, College of Medicine and Health Sciences, The University of Gondar, Gondar, Ethiopia; 2 Department of Environmental and Occupational Health and Safety, Institute of Public Health, College of Medicine and Health Sciences, The University of Gondar, P.O.Box 196, Gondar, Ethiopia; 3 Department of Epidemiology and Biostatistics, School of Public Health, College of Medicine and Health Sciences, The University of Bahirdar, P.O.Box 79, Bahirdar, Ethiopia

**Keywords:** Tuberculosis, HIV Infection, Incidence, Predictors, Gondar

## Abstract

**Background:**

Tuberculosis (TB) is the leading killer of people living with HIV (PLHIV). Many of these deaths occur in developing countries. This study aimed at determining the incidence and predictors of tuberculosis among PLHIV.

**Methods:**

A five year retrospective follow up study was conducted among adult PLHIV. The Cox proportional hazards model was used to identify predictors.

**Results:**

A total of 470 patients were followed and produced 1724.13 Person-Years (PY) of observation, and 136 new TB cases occurred during the follow up period. The overall incidence density of TB was 7.88 per 100 PY. It was high (95.9/100PY) in the first year of enrolment. The cumulative proportion of TB- free survivals was 79% and 67% at the end of the first and fifth years, respectively. Baseline WHO clinical stage III (AHR = 2.88, 95% CI = 1.53-5.43), WHO clinical stage IV (AHR = 3.82, 95% CI = 1.86-7.85), CD4 count <50 cell/ul (AHR = 2.13, 95% CI = 1.28-3.53) and ambulatory or bed ridden functional status (AHR = 1.64, 95%CI = 1.13-2.38) were predictors of time to TB occurrence.

**Conclusions:**

TB incidence rate among PLHIV, especially in the first year of enrollment was high. Advanced WHO clinical stage, limited functional status, and low CD4 count (<50 cell cell/ul) were found to be the independent predictors of TB occurrence. Early care seeking and initiation of HAART to improve the CD4 count and functional status are important to reduce the risk of TB infection.

## Background

TB is a major public health problem and the most frequently diagnosed opportunistic infection among PLHIV worldwide [1,2]. It is still the leading killer of PLHIV causing one in four deaths mostly in developing countries [3]. Worldwide, about 11.1 million adults most of whom live in Sub-Saharan Africa are co-infected [4].

The main reason for the resurgence of TB in Africa is not the deterioration of control programs; it is rather the link between TB and HIV/AIDS in addition to the lack of adequate economic and human resources. People with a latent infection of mycobacterium tuberculosis (MTB), which accounts for about one-third of the inhabitants of Sub-Saharan Africa, are at a greater risk of developing active TB if they are also immunologically weakened by concurrent HIV infection [5]. In HIV infected individuals, the presence of TB increases the occurrence of other opportunistic infections and HIV replication that lead to an increased viral load which in turn results in a more rapid progression of HIV disease. One third of all PLHIV worldwide are latently infected with MTB, making them 21–34 times more likely to develop active TB than people who are HIV negative [5-7]. Late TB diagnosis contributes to increased death rates among PLHIV [4]. The management of a TB and HIV co-infected individual is challenging because of pill burden [8], increased drug adverse effects [9], drug interaction [10] and the immune reconstitution inflammatory syndrome (IRIS) [11].

In Ethiopia, TB/HIV co-infected individuals have a greater risk of common mental disorders, low quality of life, and poor physical health than HIV infected individuals without active TB [12,13]. In recent years, great efforts have been made to integrate TB diagnosis and treatment with HIV care in order to prevent, diagnose and treat TB among PLHIV. This needs an adequate understanding of the situation through additional research in the study area where TB incidence rate and associated factors are not yet studied. This study, therefore, aimed at determining the incidence and predictors of TB among PLHIV who attended ART care at Gondar University Referral Hospital.

## Methods

### Study design and setting

A five-year institution based retrospective follow up study was conducted at the University of Gondar Referral Hospital, a teaching hospital in Gondar town, northwest Ethiopia. The hospital serves more than five million people of North Gondar and neighboring zones. The HIV care service in this hospital was initiated in 2005 with three clinics: The Adult Anti-Retroviral Therapy (ART) Clinic, Pediatric ART Clinic, and VCT Clinic. Currently, more than 7,000 patients are in active follow-up, of which over 3,888 have been initiated on highly active antiretroviral treatment (HAART). The hospital uses standardized monitoring and evaluation tools, and the data collection and management processes are well controlled and supported by electronic data back-up and processing. Patient data, including socio-demographic characteristics, like age, sex, residence, family size, level of education, occupation, marital status, substance use, and disclosure status are recorded on enrollment in the HIV Program. The patient database also includes information on WHO clinical stage, CD4 counts, Haemoglobin level (Hgb), and HAART.

The treatment is given based on the national HAART guidelines of the Ethiopian Federal Ministry of Health which consists of daily cotrimoxazole prophylaxis for patients in clinical stage 2, 3 or 4 ( irrespective of CD4 count) or CD4 <350 irrespective of WHO clinical stage. After ruling out active TB, isoniazid prophylaxis is considered in order to protect the PLHIV from developing TB disease. HAART is initiated when the CD4 count < 200 cells/mm3 at any WHO clinical stage or WHO clinical stage 4 (no matter what the CD4 count) or WHO clinical stage 3 (if the CD4 count < 350 cells/mm3). First-line HAART consists of two non-nucleoside reverse transcriptase inhibitor stavudine (d4T) or zidovudine (AZT) in combination with lamivudine (3TC) plus a non-nucleoside reverse transcriptase inhibitor in standard doses (nevirapine (NVP) or efavirenz (EFV)). The choice of HAART combination is up to decision of the physician and the availability of the drug at the time of initiation.

### Definitions of tuberculosis

TB is diagnosed using microscopic examinations of sputum smears, chest radiology, and fine-needle aspiration of lymphadenopathy and cytology with very high clinical grounds. No mycobacterial culture facilities are available in the hospital. When patients are diagnosed with active TB, the treatment is given according to the National TB Program recommendations with a standard 8 month regimen with an 8 week, 4 drug (isoniazid, rifampicin, ethambutol and pyrazinamide) intensive phase and a subsequent 6 month, 2 drug (isoniazid, ethambutol) continuation phase. Incident TB, which is defined in this study, as an event, during follow-up was ascertained retrospectively when the patient is diagnosed for TB and starts anti TB treatment. PLHIV who were not diagnosed for TB until the end of the follow up period were considered as censored.

### Inclusion and exclusion criteria

All PLHIV aged 15 years and above and were newly enrolled into the adult chronic HIV care clinic at the University of Gondar Referral Hospital from September 11, 2006 to August 31, 2007, were included in the study and followed for five years, until February 29, 2012. This period was selected in order to have the nearest five year follow up study period. In this period the facility started full implementation of standardized formats, documentation, and a recording system in a regular manner. A total of 529 PLHIV were registered during the period out of whom 59 patients were excluded due to missing charts or incomplete baseline and follow-up data.

### Data collection

All available information on patient records was checked and an appropriate data extraction format was prepared. Then, data were extracted from patients’ charts by four nurses who had ART training and experience in HIV care.

### Data analysis

Data were cleaned and entered into a computer using EPI info version 3.5.3 statistical software and exported to the Statistical Package for Social Science (SPSS) version 20 for analysis. Summary statistics and Incidence Density Rate (IDR) were calculated. To calculate TB incidence among PLHIV, the total duration of follow-up for the whole cohort in PY was used. The duration of follow-up for PLHIV who did not develop TB was calculated from the time of enrolment in the HIV Care Program until the last visit. For these people, the total duration of follow-up was considered TB-free. For PLWH who developed TB, the TB-free follow-up was calculated from the time of enrolment in the HIV Care Program until the development of TB. Subsequently, the number of TB cases within the cohort was divided by the TB-free follow-up duration and reported per 100 PY.

As this study has considered time-to-event data, the survival analysis technique was carried out, Cox proportional hazards model was fitted, and a life table was used to estimate cumulative probabilities. The Kaplan-Meier curve (an intuitive graphical presentation which describes survivorship of the study population) was used to estimate the median duration of TB occurrence. The Log rank test was used to compare survival curves between different categories of explanatory variables. Bivariate and multivariate Cox proportional hazard models were used to identify the predictors of TB occurrence. Variables with p value < 0.2 in the bivariate analysis were entered into the multivariate proportional hazard model. Hazard Ratios (HR) with 95% confidence intervals were computed and statistical significance was accepted at the 5% level (p < 0.05). The necessary assumptions for Cox proportional hazard model were checked using the Schoenfield residuals test.

### Ethical issues

Ethical clearance was obtained from the institutional review board of the Institute of Public Health, the University of Gondar. Letter of permission was obtained from the Chief Executive Officer of the hospital. The department head of the HIV care clinics gave the consent for extracting data from records. Patient names and identification numbers were not extracted so as to ensure confidentiality of patient information.

## Results

Four hundred seventy records of PLHIV were analyzed. Their mean age was 33.22 (± 7.8 SD) years and almost half, 222 (47.2%), of them were in the age group of 25–34 years. Over half (61.9%) of the PLHIV were females and the majority (87.7%), of them were urban dwellers. A total of 364 (77.4%) patients disclosed their HIV status, to their brothers/sisters (29.8%), to parents (25.3%). Three hundred eight patients (67.7%) were addicted to alcohol (26.8%), to tobacco (9.4) or to drugs 17 (3.6%) (Table 1). The eligibility criterion for initiation of HAART was mainly the WHO Clinical Stage in 282(60.0%). More than half (62.6%) of them were at WHO clinical stage 3 during enrolment. Three hundred forty two (72.8%) of the participants were on working functional status at baseline. The median CD4 count during enrollment and end of follow up was 135 [IQR: 70–207] and 335[IQR: 225–478] cells per mm^3^, respectively. The predominant regimens initially prescribed were a combination of zidovudine, Lamivudine and Nevirapine (38.3%), followed by Stavudine, Lamivudine and, Nevirapine (26.8%). One hundred eighty four (39%) patients had changed their initial regimen during the follow up period mainly to a combination of zidovudine, Lamivudine and Nevirapine (1c) 45(9.6%). Nine (1.9%) patients were switched to second line HAART. For 34(7.2%) and 29(6.1%) patients, regimens were changed due to drug side effect and TB, respectively while the reasons for changing the initial regimen were not recorded for 99 (21.1%) patients. Half of the HAART regimens were changed within the first year of follow up (Table 2).

**Table 1:** **Baseline socio demographic and clinical characteristics of PLHIV on chronic HIV care at University of Gondar Referral Hospital, September 2006 to February**, **2012**

**Characteristics**	**Number**	**Percent**
Age		
**15**-**24**	47	10.0
**25**-**34**	222	47.2
**35**-**44**	154	32.8
≥ **45**	47	10.0
Sex		
**Male**	179	38.1
**Female**	291	61.9
Marital Status		
**Single**	78	16.6
**Married**	178	37.9
**Separated**/**divorced**	144	30.6
**Widowed**	70	14.9
Religion		
**Orthodox**	431	91.7
**Muslim**	34	7.2
**Protestant**	5	1.1
Level of Educational		
**No education**	127	27.0
**Primary**	135	28.7
**Secondary**	138	29.4
**Tertiary**	70	14.9
Occupation		
**Employed**	94	20.0
**Unemployed**	376	80.0
Address		
**Urban**	412	87.7
**Rural**	58	12.3
Disclosure status		
**Disclosed**	364	22.6
**Not Disclosed**	106	77.4
Addiction		
**Addicted**	318	67.7
**Not Addicted**	152	32.3
Family size		
≤ **2**	126	26.8
**3**-**4**	169	35.9
≥**5**	175	37.2
ART eligibility criteria		
**WHO clinical stage**	282	60.0
**CD4 count**	38	8.1
**Both**	117	24.9
**Not Recorded**	33	7.0
Initial regimen		
**1a**	126	26.8
**1b**	56	11.9
**1c**	180	38.3
**1d**	97	20.6
**Others** *	11	2.3
Regimen change during follow up		
**Yes**	184	39.1
**No**	283	60.2
New regimen		
**First line**	175	37.1
**2**^**nd **^**line**	9	1.9
Reason for switch first regimen		
**Side effect**	34	7.2
**Pregnancy**	6	1.3
**Tuberculosis**	29	6.2
Others**	16	3.4
**Not recorded**	99	21.1
Past TB treatment history		
**Yes**	153	32.5
**No**	317	67.5
Functional status		
**Working**	342	72.8
**Ambulatory**	110	23.4
**Bed redden**	18	3.8
WHO clinical stage		
**I**	25	5.3
**II**	77	16.4
**III**	294	62.6
**IV**	74	15.7
CD4 count		
<**50**	77	16.4
**50**-**100**	100	21.3
**101**-**200**	169	36.0
**200**	124	26.4
Hgb		
<**10**	48	10.2
≥ **10**	422	89.8

**Table 2:** **Tuberculosis incidence density rate stratified by socio**-**demographic and clinical characteristics of PLHIV on chronic HIV care at University of Gondar Referral Hospital**, **September 2006 to February**, **2012**

**Characteristics**	**Total**	**PY**	**TB**	**TB IDR**
**Age** (**years**)				
15-24	47	180.20	13	7.21
25-34	222	810.68	64	7.89
35-44	154	569.84	45	7.89
≥ 45	47	163.41	14	8.56
**Sex**				
Male	179	594.82	64	10.76
Female	291	1129.31	72	6.37
**Marital status**				
Never married/single	78	280.87	24	8.54
Married	178	652.79	51	7.81
Separated/divorced	144	526.85	40	7.59
Widowed/er	70	263.61	21	7.96
**Religion**				
Orthodox	431	1585.37	126	7.94
Muslim	34	119.71	9	7.52
Protestant	5	19.05	1	5.23
**Educational status**				
No education	127	501.87	31	6.17
Primary	135	464.72	46	9.89
Secondary	138	510.56	38	7.44
Tertiary	70	246.98	21	8.50
**Occupation**				
Employed	94	351.48	24	6.83
Unemployed	376	1372.65	112	8.16
**Residence**				
Urban	412	1515.84	122	8.05
Rural	58	208.29	14	6.72
**Disclosure Status**				
Disclosed	364	1303.46	110	8.44
Not Disclosed	106	420.67	26	6.18
**Addiction**				
Addicted	318	1186.85	89	6.83
Not Addicted	152	537.28	47	8.74
**Total**	470	1724.13	136	7.89
**Initial regimen**				
1a	126	487.06	32	6.57
1b	56	145.15	28	19.29
1c	180	715.15	38	5.31
1d	97	332.51	34	10.22
Others^*^	8	30.28	4	13.21
**Past TB treatment history**				
Yes	153	540.89	54	9.98
No	226	859.10	58	6.75
**Functional status**				
Working	342	1342.08	79	5.88
Ambulatory	110	331.91	48	14.46
Bed ridden	18	50.2	9	17.93
**WHO clinical stage**				
I	25	105.38	3	2.84
II	77	340.55	8	2.35
III	294	1073.84	92	8.56
IV	74	204.37	33	16.14
**CD4 count**				
<50	77	226.15	36	15.91
50-100	100	364.10	25	6.86
101-200	169	640.07	48	7.49
>200	124	493.80	27	5.46
**Hgb**				
<10	48	161.30	16	9.92
≥ 10	422	1562.83	120	7.68
**Year of follow up**				
≤ 1	98	98	94	95.92
1-3	20	44.08	15	34.02
≥ 3	352	1664.79	27	1.62

### Tuberculosis incidence density

**Figure 1:**
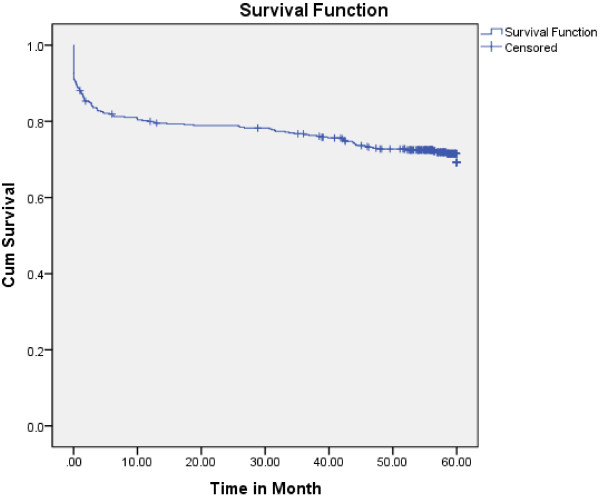
**Kaplan-Meier curve of TB survival proportion for PLHIV on Chronic HIV care at the University of Gondar Referral Hospital, September 2006 to February, 2012.**

Four hundred seventy study participants who were followed for different periods in five years produced 1724.13 PY of observation. Within the follow up period, 136 new TB cases were observed. The overall TB incidence density was 7.89 per 100 PY. Among the new TB cases, 64 were males and 72 females. Of these, 81(17.2%) were pulmonary TB and 18(3.8%) were Extra-pulmonary and/or Disseminated TB. Ninety four (69.11%) of the TB cases occurred within the first year of follow up. The highest incidence of TB was observed in the first year of enrolment (95.9/100 PY) and then decreased in the subsequent years of follow up (34.0 and 1.6 per 100 PY in three and five years, respectively). The cumulative probability of TB-free survival at the end of 6 months was 0.80; where surviving at the end of one year was 0.79; at the end of two years 0.78; at the end of three years 0.76; at the end of four years 0.72 and at the end of five years 0.67. The median survival time from enrolment to chronic HIV care to TB occurrence was 60 month (Figure 1).

### Predictors of time to TB occurrence

**Figure 2:**
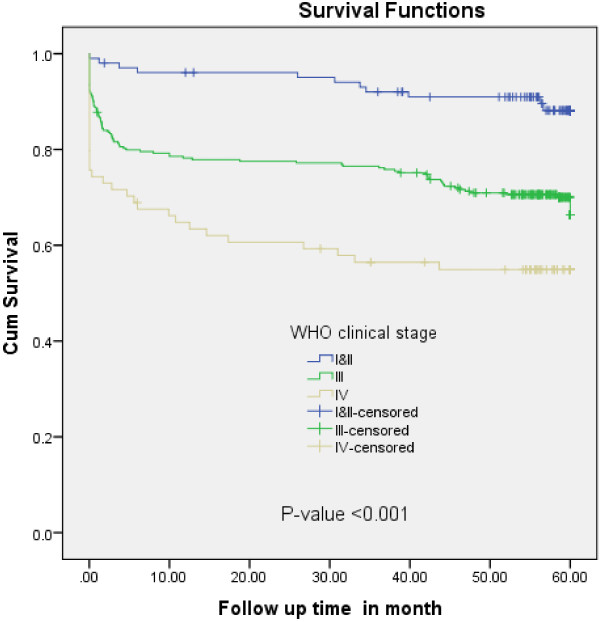
**Kaplan-Meier survival curve of TB-free proportion based on WHO Clinical Stage among PLHIV on chronic HIV care at University of Gondar Referral Hospital, September 2006 to February, 2012.**

**Figure 3:**
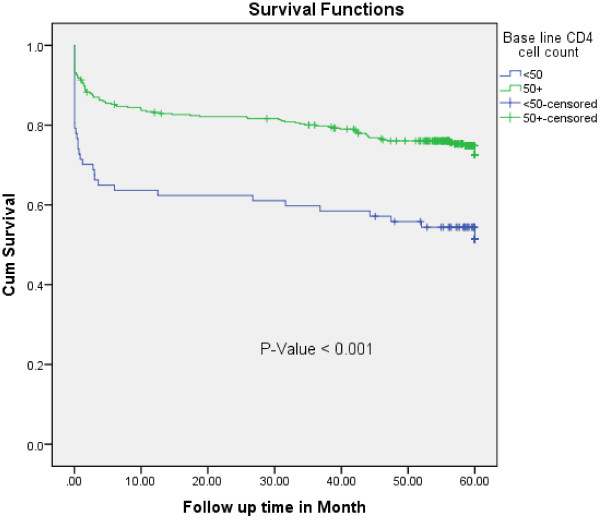
**Kaplan-Meier curve of Tuberculosis -free survival proportion based on baseline CD4 cell count at enrolment at the University of Gondar Referral Hospital from September 2006 to February, 2012.**

In the multivariate Cox-regression analysis, baseline CD4 cell count, WHO clinical stage and functional status remained significant predictors of TB- free survival time. Accordingly, PLHIV who were ambulatory or bed ridden at enrollment, were 1.64 times at higher risk of developing TB at any time compared to those who were working (AHR = 1.64, 95%CI = 1.13-2.38). The PLHIV who were at WHO clinical stage III had about three times higher risk of acquiring TB at any time compared to those with WHO clinical stage I or II (AHR = 3.26, 95%CI = 1.74-6.10). Similarly, PLHIV with WHO clinical stage IV were about four times at higher risk of TB acquisition at any time than those with WHO clinical stage I or II (AHR = 3.82, 95%CI 1.86-7.85) (Figure 2). A patient with a CD4 count of less than 50 cell/ul is 2.47 times more likely to have TB at any time than a patient with a CD4 count greater than 200 cell/ul (AHR = 2.47,95% CI 1.49-4.09) (Figure 3).

## Discussion

HIV positive people are 20 times at higher risk of developing TB as compared to HIV negative people in countries with a generalized HIV epidemic [14]. TB is also the most common cause of death among PLHIV [3]. In this study, the overall incidence of TB was 7.89 per 100 PY. It was similar with that of studies done in Ethiopia and many other Sub Sahara African countries with reported incidences ranging from 5.4 to 11 per 100 PY [15-18]. But, this finding is higher than that of studies done in Brazil and Taiwan which were 3.8% and 4.01/100 PY, respectively. This could be explained by the fact that unlike these two countries, Ethiopia is one of the high TB burden countries [2]. Studies also revealed that in very high burden countries the incidence of TB among individuals on HAART was high [19].

The highest incidence of TB was observed in the first year of enrolment. This observation was in line with the evidence of declining TB incidence after enrolment and HAART initiation reported in cohorts of HIV-infected persons in Sub-Saharan Africa [20] and Ethiopia [17]. The Peak TB incidence shortly after enrollment may have several explanations. Firstly, it may represent the progression of a subclinical disease that remained undetected during enrollment and a rapid progression of either a newly reactivated disease or an exogenous infection. Secondly, immune reconstitution inflammatory syndrome (IRIS) may have been responsible for some of these cases. TB is the most frequently reported IRIS associated infection [21]. Though the available data did not permit making this distinction, it was known that IRIS occurred in the early initiation of HAART [22].

The result of this study showed that large numbers of patients started HAART within the first year of follow up which led to the development of IRIS. The finding also showed a significant decrease in TB incidence in the subsequent years of follow up. These indirectly indicated that the reduction in TB incidence following the introduction of HAART can be particularly linked with HAART induced prevention. A study done at Arba Minch Hospital, Ethiopia, showed that TB incidences were 3.7 and 11.1 per 100 PY in the ART and Pre ART groups, respectively [23]. Other findings [17,24,25], revealed that HAART reduces the risk of TB in line with our study. Thirdly, the other possible reason for increased TB- free survival within the duration of enrolment could be the result of progressive increase in CD4 cell count which builds the immune system and may decrease the viral load over time. Although TB risk has decreased with time following enrolment, it was still significantly higher than that of the general population of Ethiopia, even after 5 years of follow up. In this study, the incidence of TB at the end of the follow up period was 1.6 per 100 PY, which was four fold higher than the overall TB rate in Ethiopia which was approximately 0.38/PY [14]. This might be due to the synergy between TB and HIV co-infection.

In the analysis, predictors significantly associated with TB-free survival time were bedriddenness or ambulatory functional status, advanced WHO clinical stage, and low CD4 count at baseline (Table 3). All these predictors were also identified in previous studies [16,18,26]. WHO clinical stage III had 3 times higher risk of TB incidence at any time than WHO clinical stage I or II. Furthermore, the risk is five times higher in WHO clinical stage IV which is in line with a study done in Nigeria [16]. Even though TB can occur at any WHO clinical stage it is more common in advanced clinical stages [6]. This research has also found that patients with ambulatory or bed ridden baseline functional status were 1.45 times at higher risk of developing TB than those with working functional status. This could be due to the fact that patients become bed ridden or ambulatory as a result of many infectious diseases as their CD4 cell count decrease. The baseline CD4 count of <50 cells/ μl was a very strong and independent risk factor of TB in patients enrolled to chronic HIV care (AHR = 2.13, 95% CI = 1.28-3.53).A lower baseline CD4 count before initiation of HAART has consistently been indicated as an independent risk factor for the occurrence of TB during the course of HIV treatment and care in different settings [16,18,26]. A study in West Africa indicated that a baseline CD4 count had no association with the occurrence of TB during HAART [27]. That was because the study had limitations in design relating to the size of the study population, the number of TB cases, diagnostic criteria for tuberculosis, and restricted cohort composition.

**Table 3:** **Cox regression analysis of predictors of tuberculosis among PLHIV cohorts on chronic HIV car at University of Gondar referral Hospital from September 2006 to February**, **2012**

**Variable**	**Survival status**		**Total**	**CHR**, **(95%CI)**	**AHR**, **(95% CI)**
	**Event (TB)**	**Censored**			
**Sex**					
Male	64	115	179	1.58 (1.13-2.22)	1.4 (0.99-1.98)
Female	72	219	291	1.00	1.00
**Tobacco use**					
Yes	18	26	44	1.64(1.00-2.70)	1.46(0.85-2.49)
No	118	306	424	1.00	1.00
**Functional status**					
Working	79	263	342	1.00	
Ambulatory/Bed ridden	57	71	128	2.27(1.61 3.19)	**1**.**64**(**1**.**13**-**2**.**38**)
**WHO clinical stage**					
I/II	91	11	102		1.00
III	202	92	294	3.26(1.74 ,6.10)	**2**.**88**(**1**.**53**-**5**.**43**)
IV	41	33	74	5.55(2.80, 0.99)	**3**.**82**(**1**.**86**-**7**.**85**)
**CD4 cell count**					
<50	41	36	77	2.61(1.58-4.30)	**2**.**13**(**1**.**28**-**3**.**53**)
50-200	196	73	269	1.28(0.82,1.99)	1.27(0.81-1.98)
>200	97	27	124	1.00	1.00

The main limitation of our study was the retrospective nature of the cohort. The study participants whose charts were lost were not included in the study, perhaps undermining the result if the charts excluded were related to TB.

## Conclusion

Incidence of TB was high among people living with HIV, especially in the first year of enrollment in chronic HIV care. WHO clinical stage III and IV, ambulatory or bed ridden functional status, and low CD4 count (<50 cell/ul) were found to be independent predictors of TB-free survival time. Therefore, it is important to give special attention to PLHIV in diagnosing, preventing and treating TB early. TB/HIV collaborative long term surveillance programs should be strengthened at chronic HIV care clinics with early initiation of HAART as recommended in the national guideline.

## Competing interest

The authors declare that they have no conflict of interests.

## Authors’ contribution

KA wrote the proposal, participated in data collection, analyzed the data and drafted the paper. BW and AN approved the proposal with some revisions, participated in data collection and analysis. All authors participated in the preparation of the manuscript and approved the final manuscript.

## Pre-publication history

The pre-publication history for this paper can be accessed here:

http://www.biomedcentral.com/1471-2334/13/292/prepub
